# Identification of Crucial Polymethoxyflavones Tangeretin and 3,5,6,7,8,3′,4′-Heptamethoxyflavone and Evaluation of Their Contribution to Anticancer Effects of *Pericarpium Citri Reticulatae ‘Chachi’* during Storage

**DOI:** 10.3390/antiox11101922

**Published:** 2022-09-28

**Authors:** Yexing Tao, Qian Yu, Yuting Huang, Ruiting Liu, Xiwen Zhang, Ting Wu, Siyi Pan, Xiaoyun Xu

**Affiliations:** 1 College of Food Science and Technology, Huazhong Agricultural University, Wuhan 430070, China; 2 Key Laboratory of Environment Correlative Dietology (Ministry of Education), Huazhong Agricultural University, Wuhan 430070, China; 3 Hubei Key Laboratory of Fruit & Vegetable Processing & Quality Control, Huazhong Agricultural University, Wuhan 430070, China

**Keywords:** *Pericarpium Citri Reticulatae ‘Chachi’*, polymethoxyflavones, storage periods, non-small cell lung cancer, apoptosis

## Abstract

*Pericarpium Citri Reticulatae ‘Chachi’* (PCR-C), rich in polymethoxyflavones (PMFs), has potential anticancer bioactivity and its quality will be improved during storage. However, the main factors influencing the PCR-C quality during its storage remain unclear. In this study, multivariate analysis was performed to investigate free and bound PMFs of PCR-C during storage. The anticancer effects of purified PCR-C flavonoid extracts (PCR-CF) and the important PMFs were evaluated using A549 cells. The results showed that PCR-C samples exhibited remarkable differences in free PMFs during storage, which fell into three clusters: Cluster 1 included fresh (fresh peel) and PCR-C01 (year 1); Cluster 2 consisted of PCR-C03 (year 3) and PCR-C05 (year 5); and PCR-C10 (year 10) was Cluster 3. 3,5,6,7,8,3′,4′-heptamethoxyflavone, tangeretin, and isosinensetin were identified as the most important PMFs distinguishing the various types of PCR-C according to its storage periods. Moreover, PCR-CF inhibited A549 cell proliferation and induced cell cycle arrest at G2/M phase, cell apoptosis, and ROS accumulation, and all anticancer indices had an upward tendency during storage. Additionally, tangeretin and 3,5,6,7,8,3′,4′-heptamethoxyflavone exhibited anticancer effects on A549 cells, whereas isosinensetin displayed no anticancer effect, indicating that tangeretin and 3,5,6,7,8,3′,4′-heptamethoxyflavone jointly contributed to anticancer activity of PCR-C during storage. PCR-CF and the most important PMFs killed cancer cells (A549 cells) but had no cytotoxicity to normal lung fibroblast cells (MRC-5 cells). Overall, the high quality of long-term stored PCR-C might be due to the anticancer effects of tangeretin and 3,5,6,7,8,3′,4′-heptamethoxyflavone.

## 1. Introduction

*Pericarpium Citri Reticulatae ‘Chachi’* (PCR-C, Guangchenpi in China), one of the first food materials promulgated by Chinese Ministry of Health owing to its outstanding efficacy, is a dried and matured pericarp of *Citrus reticulata Blanco* or its cultivars cultivated in Xinhui, Guangdong, China that can not only be used as a dietary supplement but also a traditional Chinese medicine [1]. PCR-C has pharmacological effects as the adjuvant therapy for indigestion, physical weakness, cough accompanied with expectoration of phlegm, and other digestive and respiratory diseases [2,3]. Long-term stored medicine refers to that materials that are stored and maintained through using corresponding methods and then could be more applicable to the traditional medicinal utilization [4]. There is a folk proverb about the aging of PCR-C which is “the longer storage periods, the better”, which reflects the effect of storage period on the market price and application value of PCR-C [5]. PCR-C contains abundant flavonoids, which are divided into two categories: flavanone glycosides such as hesperidin and polymethoxylated flavones (PMFs) such as tangeretin and nobiletin [6]. Although the contents of PMFs are quite low in PCR-C, they are characteristic bioactive components with extremely potent activities [7,8]. PCR-C quality is mainly attributed to the accumulation of specific chemical bioactive components during storage. The phenolic acids in PCR-C have been reported to exhibit an increasing trend, and the antioxidant activity of PCR-C is enhanced during storage, indicating that phenolic acids might be the main contributors to the improvement of the quality of long-term stored PCR-C [9]. One previous study has revealed that the contents of all the polymethoxyflavones (PMFs such as sinensetin, 4′,5,7,8-tetramethoxyflavone, nobiletin, tangeretin, and 5-O-desmethyl nobiletin) and their antioxidant activities increase during PCR-C storing, suggesting the positive correlation between the quality of PCR-C during storage and the PMFs contents [5]. On the contrary, one metabolomic analysis that was based on UPLC-QTOF-MS indicated that the contents of PMFs were not positively correlated with the storage periods of PCR-C [10]. The dynamic change of specific chemical bioactive components in PCR-C during storage is still contradictory due to the limited free fraction of components. Thus, the comprehensive evaluation on the dynamic changes of free and bound PMFs in PCR-C during storage is necessary. Among the chemical compounds, the main contributors to the quality of PCR-C during storage also remain largely unknown.

Lung cancers including non-small cell lung cancer (NSCLC, almost accounting for 85%) and small cell lung cancer are a prevailing oncological disease worldwide with high incidence rate and mortality rate [11]. The common treatments that include surgery, radiation, chemotherapy, and targeted therapy are based on the development stage of the cancer [12]. Although a variety of NSCLC treatment strategies have been developed in recent decades, the prognosis of patients is far from satisfactory, and the resistance, side effects, and recurrence are fairly common [13]. Therefore, developing potentially effective agents and exploring novel NSCLC therapeutic strategies are urgent. Natural products with the advantages of low cost, safety, and easy accessibility are considered to be the potentially effective anticancer drugs. The increasing evidence has demonstrated that PCR-C has antioxidant, anti-inflammatory, anti-asthmatic, neuroprotective, and antitumor activities [14,15,16]. However, during storage, the influential contributors to the anticancer effect of PCR-C remain unclear.

Therefore, it is worth investigating the dynamic change of free and bound PMFs in PCR-C during storage and explore the influential PMFs that mainly contribute to the improvement of anticancer effect of PCR-C during storage. In the present study, the multivariate statistical analysis was performed to reveal dynamic changes of free and bound PMFs and to identify the important PMFs that could distinguish PCR-C during storage. In addition, the anticancer effect of PCR-CF and the important PMFs were evaluated to screen the crucial bioactive components of PCR-CF during storage. The results of dynamic changes of free and bound PMFs and anticancer activity assay revealed that free PMFs mainly contributed to the difference and the formation of unique quality of PCR-C during storage, and the free fraction of 3,5,6,7,8,3′,4′-heptamethoxyflavone, tangeretin, and isosinensetin were the determinant PMFs for discriminating the types of PCR-C at different storage periods. The accumulation of free tangeretin and 3,5,6,7,8,3′,4′-heptamethoxyflavone during storage contributed to the improvement of the quality of PCR-C. Our findings will provide a theoretical basis for the scientific utilization, rational storage, and quality control of PCR-C.

## 2. Materials and Methods

### 2.1. Samples and Reagents

The fresh peel of *Citrus reticulata cv. ‘Chachiensis’* (Fresh) and PCR-C aged 1, 3, 5, 10 years (PCR-C01, PCR-C03, PCR-C05, PCR-C10) were obtained from Ganze Garden, Shuangshui Town, Xinhui District, Jiangmen City, Guangdong Province, China and authenticated by Hubei university of Chinese Medicine. PCR-CF at the fresh peel and aged 1, 3, 5, 10 years (Fresh-F, PCR-C01F, PCR-C03F, PCR-C05F, PCR-C10F) were obtained from our laboratory according to our previous study [17]. The flavonoid standards tangeretin, isosinensetin, and 3,5,6,7,8,3′,4′-heptamethoxyflavone were purchased from Yuanye Bio-Technology Co., Ltd. (Shanghai, China). Fetal bovine serum (FBS) was purchased from Germini (Woodland, CA, USA). Dulbecco’s Modified Eagle Medium/Nutrient Mixture F-12 (DMEM/F12) was obtained from HYcezmbio Biotechnology Co., Ltd. (Wuhan, China). The 3-(4,5-dimethylthiazol-2-yl)-2,5-diphenyltetrazolium bromide (MTT) was provided by Gen-View Scientific Inc. (Calimesa, CA, USA). Trypsin was obtained from Lanheng Shidai Biotechnology Co., Ltd. (Wuhan, China). Penicillin-Streptomycin Solution was purchased from Cienry Biotechnology Co., Ltd. (Huzhou, China). Annexin V FITC/PI apoptosis and cell cycle staining kit were obtained from Multi-sciences (Lianke) Biotechnology Co., Ltd. (Hangzhou, China). 2′,7′-dichlorofluorescein diacetate (DCFH-DA) and Dimethyl sulfoxide (DMSO) in cell culture grade were obtained from Sigma Chemical Co. (St. Louis, MO, USA).

### 2.2. Cell Culture

Human non-small lung cancer A549 cells and human normal fetal lung fibroblast cell line (MRC-5) were purchased from the Cell bank of Chinese Academy of Sciences in Shanghai, China and cultured in DMEM/F-12 medium that was supplemented with 10% FBS, penicillin (100 U/mL), and streptomycin (100 µg/mL) under a stable atmosphere of 5% CO_2_, at 37 °C constant temperature and a relatively high humidity of 95%. When they grew to approximately 80–90% confluence, the cells were passaged until the logarithmic growth phase and used for subsequent experiments.

### 2.3. MTT Assay

During the logarithmic growth phase, A549 cells and MRC-5 cells were grown in 96-well plates at a density of 3000 cells/well overnight. Then, the cells were treated with the indicated PMFs and PCR-CF diluted in DMEM/F12 culture medium. After 24-h treatment, 0.5 mg/mL MTT solution was added and incubated for 2–4 h. The medium was removed, and 200 uL DMSO was added to each well, followed by 10-min incubation. After shaking for 5 min at medium speed, the absorbance at 490 nm was measured using a microplate reader (MultiScan Go, Thermo Scientific Co., Ltd., Waltham, MA, USA). The cell viability percentage (%) was determined by OD treatment group/OD control group × 100%. IC_50_ was calculated by the Logit method using GraphPad Prism 7.0.

### 2.4. Cell Cycle Analysis

A549 cells were grown in 12-well plates at a density of 1 × 10^5^ cells/well overnight and treated with PMFs and PCR-CF for 24 h. The cells were harvested and fixed with ethanol (pre-cooled overnight at −20 °C), followed by centrifugation (1000 rpm, 3 min). Subsequently, the cells were stained with 1 mL DNA staining solution (PI containing RNase A, Lianke Biotech, CCS012; Hangzhou, China) for 30 min. The red fluorescence was detected by CytoFLEX S flow cytometer (Beckman Coulter) at the excitation wavelength of 488 nm within one hour and analyzed by FlowJo software 7.6.

### 2.5. Cell Apoptosis Analysis

The A549 cells were grown in 12-well plates at a density of 1 × 10^5^ cells/well overnight and treated with PMFs and PCR-CF for 24 h. The cells were washed with precooled PBS twice and harvested. After being resuspended in 500 µL binding buffer (1×) and diluted with PBS, the cells were double stained with 5 µL annexin V-FITC and 10 µL PI in the cell suspension for 5 min using the Annexin V-FITC/PI apoptosis kit (Lianke Biotech, AP101). At least 10,000 living cells were analyzed on a CytoFLEX S flow cytometer (Beckman Coulter) within one hour. The green fluorescence of annexin V-FITC was detected by the FITC channel (Ex = 488 nm, Em = 530 nm) and the red fluorescence of PI was detected by the PI channel (Ex = 535 nm, Em = 615 nm). The apoptosis rate was analyzed by FlowJo software 7.6.

### 2.6. Detection of Intracellular ROS Accumulation

The A549 cells were seeded in 12-well plates at a density of 1 × 10^5^ cells/well overnight and treated with PMFs and purified PCR-CF for 24 h. Afterwards, the cells were incubated for 45 min at 37 °C and washed with PBS twice. After DCFH-DA (10 μM) staining, intracellular ROS accumulation was detected by flow cytometry. The fluorescence intensity was measured by a flow cytometer at an excitation wavelength of 488 nm and an emission wavelength of 525 nm within one hour. The ROS level was analyzed by FlowJo software 7.6.

### 2.7. Data Processing and Statistical Analysis

All the data were expressed as the means ± SD (standard deviation) of the triplicates (*n* = 3) from independent experiments. The data were processed using GraphPad Prism 7.0 (GraphPad Software Inc., La Jolla, CA, USA). The IC_50_ values were obtained from nonlinear regression. One way ANOVA analysis and subsequent Duncan’s test were performed for comparison among the samples, and *p* < 0.05 was considered as statistically significant. The hierarchical clustering analysis (HCA) was performed by TBtools software. Principal component analysis (PCA) was conducted using Origin software (version 2021; Origin Lab, Northampton, Massachusetts, USA). PLS-DA analysis was performed by SIMCA software (version 14.1).

## 3. Results and Discussion

### 3.1. Multivariate Statistical Analysis of Bound and Free PMFs during PCR-C Storage

The bound and free PMFs in positive and negative ion modes during PCR-C storage were examined qualitatively and quantitatively by HPLC-Q-TOF-MS/MS based on our previous research, and the contents of eight PMFs with high response intensity including isosinensetin (C1), sinensetin (C2), 5,7,8,4′-tetramethoxyflavone (C3), nobiletin (C4), 5,6,7,4′-tetramethoxyflavone (C5), 3,5,6,7,8,3′,4′-heptamethoxyflavone (C6), tangeretin (C7), and 5-demethylnobiletin (C8) were determined by HPLC [17].

**Figure 1:**
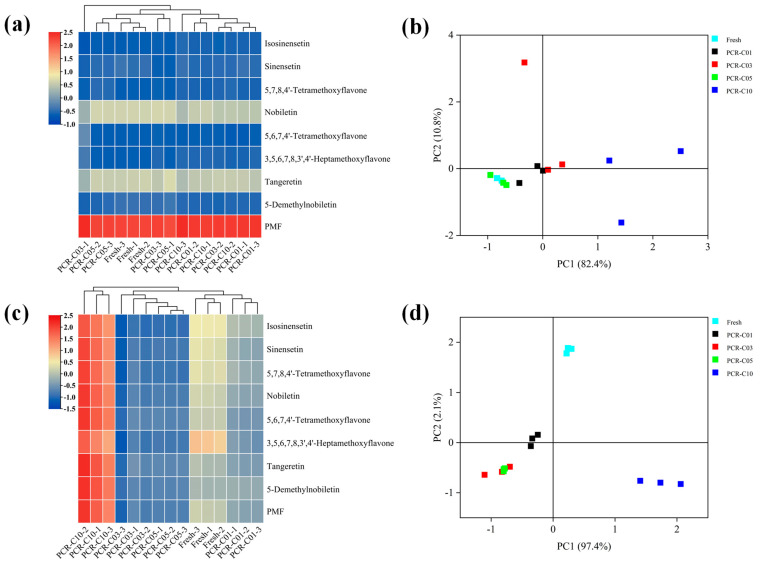
Multivariate statistical analysis of the PMFs in the PCR-C during storage. (**a**) HCA and (**b**) PCA of the bound PMFs in PCR-C (Fresh, PCR-C01, PCR-C03, PCR-C05, PCR-C10); (**c**) HCA and (**d**) PCA of the free PMFs in PCR-C (Fresh, PCR-C01, PCR-C03, PCR-C05, PCR-C10).

The multivariate statistical analysis was performed to investigate the dynamic changes and the contribution of these eight main PMFs to the diversity of the PCR-C at different storage periods. A cluster heatmap of the bound and free PMFs were plotted, respectively. As shown in Figure 1a, with the extended storage periods, the bound PMFs in PCR-C at different storage periods were not clearly clustered, which indicated that the bound PMFs were similar without significant differences during PCR-C aging. Then, a PCA score plot showed a similar result (Figure 1b). In contrast, Figure 1c showed that the free PMFs in PCR-C10 were clearly separated from those in other PCR-C (Fresh, PCR-C01, PCR-C03, PCR-C05), indicating a remarkable difference in the free PMFs in the late storage period (year 10) of PCR-C. In addition, Figure 1d showed that in terms of the free PMFs, PCR-C fell into three clusters: Cluster 1 was fresh and PCR-C01; Cluster 2 included PCR-C03 and PCR-C05; and Cluster 3 was PCR-C10. And as shown in Figure 1c, with the extended PCR-C storage time, the contents of the free PMFs from Cluster 1 to Cluster 3 exhibited an increasing trend. Thus, it could be concluded that the free PMFs in PCR-C varied with the storage periods, and they mainly contributed to the difference and the formation of unique quality of PCR-C during storage. The contents of the free PMFs had an increasing trend with the extended storage periods of PCR-C. The above results demonstrated that the free PMFs were markedly affected by the storage periods of PCR-C.

Our results were consistent with one previous report that the contents of the 15 types of flavonoid metabolites in PCR-C (year 0, 1, 2, 3, 4, and 29) were increased with the extended storage periods [18]. In addition, the contents of the free phenolic acids in 3-year PCR-C clearly differed from those at 1-year PCR-C [19]. When the whole phenolics profile including phenolic acids and flavonoids are taken into consideration, the free fraction showed a similarity and a slight variation between PCR06 and PCR13. On the contrary, the bound fractions in PCR-C at the later stored period (year 13) were distinct from those in the early time of PCR06, PCR03, and fresh [20]. These previous findings were not in line with our study results, and the possible reason for such an inconsistency might be that we mainly investigated the dynamic changes of PMFs rather than the entire phenolics profiles.

### 3.2. Screening of Important PMFs in PCR-C during Storage

The clustering analysis and PCA successfully divided the PCR-C in different storage periods into three clusters in terms of the free PMFs. In order to screen the potential chemical biomarkers that could distinguish the three clusters of PCR-C, the partial least squares linear discriminant analysis (PLS-DA) was performed to establish the discriminatory models [21]. One previous study has shown that *Citrus reticulata “Chachi”* and *Citrus reticulata Blanco* samples could be separated from each other based on the GC-MS data of volatile compounds by employing PCA, HCA, and orthogonal partial least-squares-discrimination analysis (OPLS-DA), and seven potential chemical markers were identified to be responsible for the special quality control of *Citri reticulatae pericarpium* (CRP) [22].

**Figure 2:**
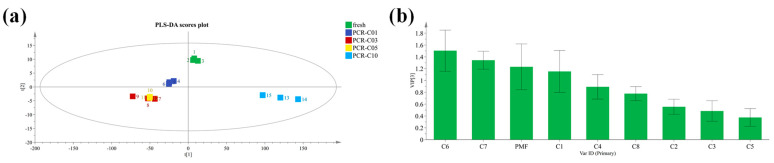
(**a**) PLS-DA of PCR-C samples and (**b**) variable importance in projection (VIP) score based on the free PMFs in PCR-C (Fresh, PCR-C01, PCR-C03, PCR-C05, PCR-C10). C1, isosinensetin; C2, sinensetin; C3, 5,7,8,4′-tetramethoxyflavone; C4, nobiletin; C5, 5,6,7,4′-tetramethoxyflavone; C6, 3,5,6,7,8,3′,4′-heptamethoxyflavone; C7, tangeretin; and C8, 5-demethylnobiletin.

Considering this, a PLS-DA regression model was established in the present study. R2X, R2Y, and Q2 of the model were 1, 0.727, and 0.659, respectively, which showed that our established model was good. As shown in Figure 2a, in terms of free PMFs, fresh was close to PCR-C01; PCR-C03 was close to PCR-C05; and PCR-C10 was an independent cluster, which was consistent with our PCA results. In the PLS-DA model, the variable importance in the projection (VIP) value was a parameter to evaluate the contributions of the variables to screening biomarkers. Generally, the variables were considered as differential biomarkers when the VIP scores were higher than 1 (VIP > 1) [23]. In our study, the VIP scores of three chemical components including 3,5,6,7,8,3′,4′-Heptamethoxyflavone (C6), Tangeretin (C7), and Isosinensetin (C1) were greater than 1 (VIP > 1) (Figure 2b), which indicated that these three compounds were important variables to discriminate the three clusters of PCR-C at different storage periods, namely, they were the determinant PMFs for discriminating the types of PCR-C at different storage periods. Our results were in line with the previous findings that phenolic acids, flavonol glycosides, fatty acids, and alkyl glycosides were identified as marker compounds by untargeted metabolomics analysis [24].

### 3.3. PCR-CF Inhibits Cell Growth of A549

Our previous study has confirmed that the content of PMFs in PCR-C that was stored for 10 years was higher than that in the PCR-C at early stored periods (fresh peel, 1, 3, and 5 years), and that PCR-C10F had excellent antioxidant activity which was positively correlated with nobiletin and 5,6,7,4′-tetramethoxyflavone [17]. Therefore, PMFs were the main active components in PCR-C, and their accumulation might contribute greatly to the efficacy of PCR-C during storage.

**Figure 3:**
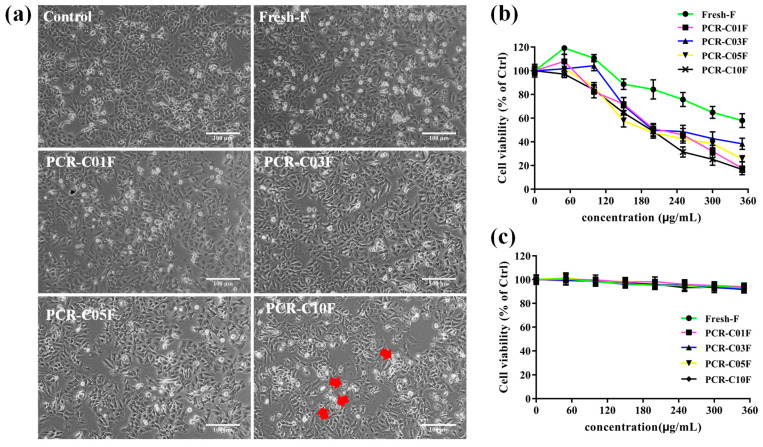
Growth inhibition effect of PCR-CF during storage on A549 cells. (**a**) Morphological changes of A549 cells that were treated with PCR-CF for 24 h under inverted phase-contrast microscopy (scale bar = 100 μm). (**b**) Viability of A549 cells and (**c**) MRC-5 cells that were treated with PCR-CF at the gradient concentrations (50, 100, 150, 200, 250, 300, and 350 µg/mL) for 24 h by MTT assay.

Since the free PMFs were the most influential factors discriminating the types of PCR-C during storage, and they showed an increasing trend, we further purified the free PMFs in PCR-C (PCR-CF) using HPD300 resin [17] to investigate their anticancer effect. Recently, A549 cells, human alveolar epithelial cells, have been widely and universally applied to investigate the anticancer effect of natural products in NSCLC [25,26,27]. Firstly, the morphology changes of A549 cells that were treated with PCR-CF at different storage periods were observed under the inverted phase-contrast microscopy. As shown in Figure 3a, morphology change in A549 cells was observed after treatment with PCR-CF at different storage periods at the dose of 200 μg/mL. With the extended storage periods, the A549 cell number was decreasing and the cell displayed a shrunken shape and detached. In addition, plasma membrane blebbing was observed when the cells were treated with PCR-C10F (indicated by arrows). To determine whether PCR-CF at different storage periods exhibited an anticancer effect against NSCLC and whether the anticancer effect was enhanced with aging years, the cell viability of NSCLC cells (A549) was detected by an MTT assay. As shown in Figure 3b, the results of the MTT assay indicated that 24-h treatment with PCR-CF at different storage periods significantly inhibited the growth of A549 cells in a dose-dependent manner and the cytotoxicity of cells that were treated with PCR-C10F was higher than those that were treated with PCR-CF at early storage periods. Notably, the inhibition effect of PCR-C10F on cell proliferation was better than that of fresh-F, PCR-C01F, PCR-C03F, and PCR-C05F at the IC_50_ values (half maximal inhibitory concentration) of 190.2 µM, 379.4 µM, 213.7 µM, 253.6 µM, and 208.2 µM, respectively (Table 1). Our results were consistent with previous reports that citrus fruit-specific flavonoids nobiletin and 5-demethylnobiletin significantly inhibited the proliferation of human non-small cell lung cancer cells [28]. The anti-proliferative effect of PCR-CF was similar to flavonoids that were isolated from Korean *Citrus aurantium* L. with 230 μg/mL IC_50_ value [29,30]. In addition, PCR-CF at different storage periods had no significant effect on the growth of MRC-5 cells (Figure 3c), indicating that PCR-CF had an inhibitory effect on the growth of cancer cells but a little effect on normal lung cells with no toxicity. These results demonstrated that PCR-CF inhibited the growth of A549 cells in vitro and exhibited an enhanced inhibitory effect when storage years was extended, which might explain why the long-term stored PCR-C had high quality.

### 3.4. PCR-CF Induces Cell Cycle Arrest in A549 Cells

**Figure 4:**
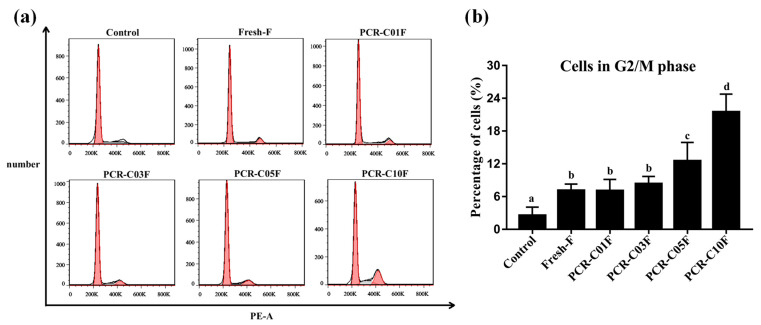
PCR-CF-induced G2/M phase cell cycle arrest in A549 cells. (**a**) The effect of PCR-CF on cell cycle distribution of A549 cells. Fluorescence intensity of A549 cells with or without 24-h PCR-CF (200 µg/mL) treatment by PI staining was measured by flow cytometry. (**b**) Percentage of cells at G2/M phase of cell cycle. The data were expressed as the means ± SD of the triplicates of each independent experiment. The different lowercase letters above the bars indicated a significant difference (*p* < 0.05) between the different groups through one-way ANOVA and Duncan’s multiple comparisons.

In order to determine whether PCR-CF inhibited cell growth via cell cycle arrest, the effect of PCR-CF at different storage periods on cell cycle arrest of A549 cells was investigated by flow cytometry with PI staining. As shown in Figure 4a, compared to the control group, the PCR-CF treatment group exhibited a significant induction on cell cycle arrest at the G2/M phase, which agreed with the previous report that tangeretin induced G2/M cell cycle arrest and apoptosis to suppress cell growth and to induce cell death in glioma cells [31]. The cell number at the G2/M cell cycle arrest phase ranked in descending order as follows: PCR-C10F > PCR-C05F > PCR-C03F > PCR-C01F > Fresh-F (Figure 4b). Such a change trend demonstrated that the cell cycle arrest that was induced by PCR-CF was enhanced during storage. The cell number at G2/M cell cycle arrest phase of PCR-C10F and PCR-C05F treatment group was significantly higher than that of the other PCR-CF treatment groups. In summary, PCR-CF inhibited cell growth by inducing G2/M phase cell cycle arrest of A549 cells and exhibited an enhanced effect with storage periods extended.

### 3.5. PCR-CF Induces Apoptosis in A549 Cells

**Figure 5:**
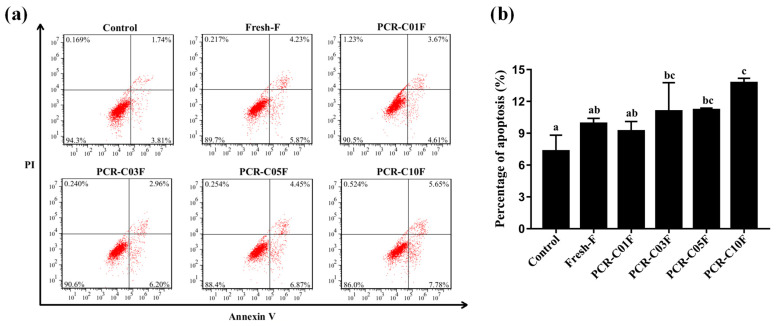
PCR-CF-induced apoptosis of A549 cells. (**a**) The effect of PCR-CF on apoptosis of A549 cells. The apoptosis rate of A549 cells with or without 24-h PCR-CF (200 µg/mL) treatment followed by Annexin V-FITC/PI double staining was measured by flow cytometry. (**b**) The apoptosis rate of A549 cells was calculated (early and late apoptosis). The data were expressed as the means ± SD of the triplicates of each independent experiment. The different lowercase letters above the bars indicated a significant difference (*p* < 0.05) between the different groups through one-way ANOVA and Duncan’s multiple comparisons.

One previous study has revealed that the ethyl acetate extracts from sweet orange peel exert an antiproliferative effect on human hepatoma cells through cell cycle arrest and apoptosis induction [32]. In order to explore whether PCR-CF induced A549 cell apoptosis, flow cytometry assay with Annexin V-FITC/PI double staining was conducted. As shown in Figure 5a, compared to the control group, the PCR-CF treatment group exhibited a significant induction of apoptosis. The percentage of apoptosis that was induced by PCR-CF treatment ranked in descending order as follows: PCR-C10F > PCR-C05F > PCR-C03F > Fresh-F > PCR-C01F (Figure 5b). Compared with the PCR-CF at early storage periods, PCR-C10F significantly induced apoptosis of A549 cells, indicating higher apoptosis induction of PCR-C10F.

Taken together, our results demonstrated that PCR-CF inhibited the cell growth by inducing cell arrest at the G2/M phase and apoptosis of A549 cells in vitro, and there was an enhanced inhibition trend with extended storage time, which could explain the high quality of the long-term stored PCR-C.

### 3.6. PCR-CF Induces ROS Accumulation in A549 Cells

**Figure 6:**
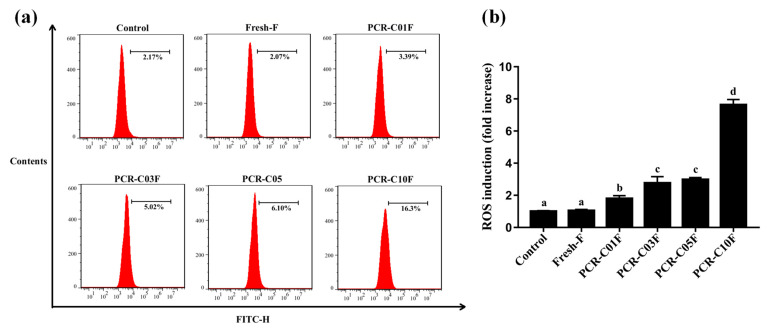
ROS accumulation that was induced by PCR-CF in A549 cells. (**a**) ROS generation in A549 cells was measured by flow cytometry after A549 cells were exposed to 200 µg/mL PCR-CF for 24 h followed by DCFH-DA staining. (**b**) Fluorescence intensity ratio of the treatment group to control group. The data were expressed as the means ± SD of the triplicates of each independent experiment. The different lowercase letters above the bars indicate a significant difference (*p* < 0.05) between the different groups through one-way ANOVA and Duncan’s multiple comparisons.

The excessive accumulation of ROS leads to the suppression of cancer angiogenesis, metastasis, and cancer cell survival [33]. Multiple chemotherapeutic agents have been reported to induce cell apoptosis and cell death through ROS accumulation [34,35]. One previous study has found that Kaempferol can inhibit the Nrf2 signaling pathway, thus inducing ROS accumulation, eventually resulting in apoptosis [36]. In order to determine whether ROS was involved in PCR-CF-induced apoptosis of A549 cells, ROS generation in A549 cells was detected by flow cytometry with DCFH-DA staining. As shown in Figure 6a,b, the level of ROS was increased with the extended storage periods, and the level of ROS in the A549 cells that were treated with the PCR-C10F was significantly higher than those that were treated with PCR-CF at early storage periods when A549 cells were treated with 200 µg/mL PCR-CF for 24 h. The ROS accumulation in A549 cells might be associated with PCR-CF-induced cytotoxicity. In conclusion, these results suggested that ROS accumulation that was induced by the PCR-CF showed an increasing trend with extended storage periods and might mediate the cell viability inhibition, cell cycle arrest, and apoptotic induction in A549 cells.

### 3.7. Important PMFs Inhibit the Cell Growth and Induce Apoptosis of A549 Cells

Since isosinensetin, 3,5,6,7,8,3′,4′-heptamethoxyflavone, and tangeretin were the important factors influencing the quality of PCR-C during storage, and they exhibited antioxidative [37,38], anti-inflammatory [39], neuroprotective [40,41], and anti-tumor [42,43,44] bioactivities according to the previous studies, this study further evaluated their anticancer effect against NSCLC (A549 cells) in vitro.

**Figure 7:**
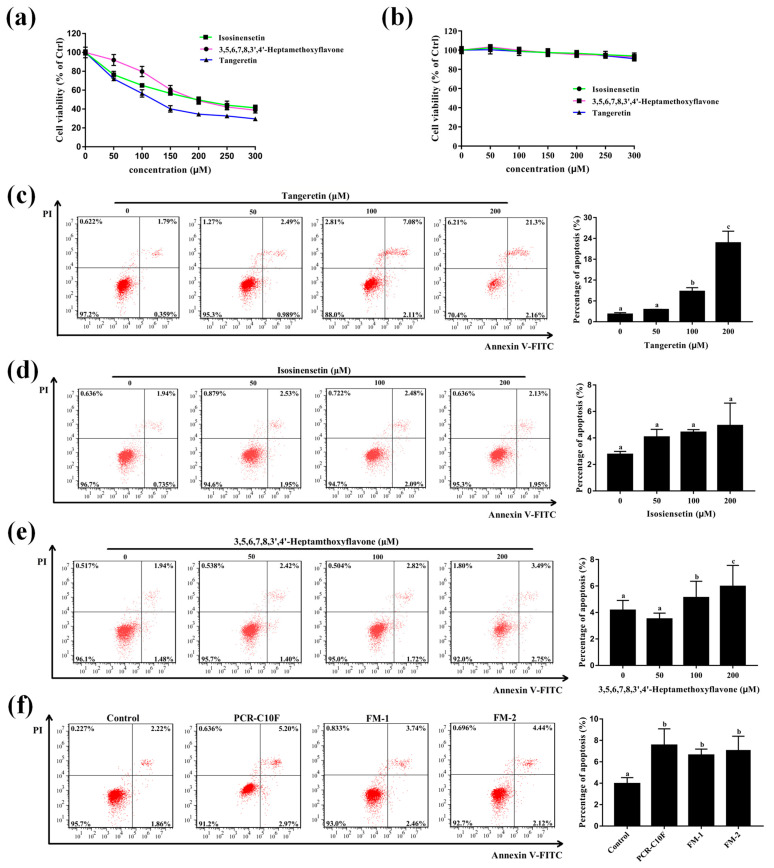
Growth of inhibition and apoptosis induction effect of the important PMFs in A549 cells. (**a**) Viability of A549 cells and (**b**) MRC-5 cells treated with the important PMFs at gradient concentrations (50, 100, 150, 200, 250, and 300 µM) for 24 h by MTT assay. The apoptosis induction effect of tangeretin (**c**), 3,5,6,7,8,3′,4′-Heptamethoxyflavone (**d**), and Isosinensetin (**e**) in A549 cells and the corresponding apoptosis rate of A549 cells (the early and late apoptosis). The apoptosis rate of A549 cells with or without 24-h PMFs (50, 100, and 200 µM) treatment followed by Annexin V-FITC/PI staining was measured by flow cytometry. (**f**) The apoptosis induction effect of PCR-C10F and the major active flavonoid mixture (FM) with the equivalent amount of PMFs to PCR-C10F. The data were expressed as the means ± SD of the triplicates of each independent experiment. The different lowercase letters above the bars indicate a significant difference (*p* < 0.05) between the different groups through one-way ANOVA and Duncan’s multiple comparisons.

To validate whether the important PMFs had an anticancer effect against NSCLC, the effect of PMFs on cell viability of NSCLC cells (A549) was detected by an MTT assay. The MTT assay results indicated that 24-h treatment with the important PMFs significantly inhibited the growth of A549 cells in a dose-dependent manner (Figure 7a). Notably, the inhibitory effect on cell growth of tangeretin was better than isosinensetin and 3,5,6,7,8,3′,4′-heptamethoxyflavone at the IC_50_ of 118.5 µM, 197.6 µM, and 208.6 µM, respectively (Table 2), indicating that they could inhibit A549 cell growth even at the low concentration, and thus they had the good anticancer effect on A549 cells. In addition, isosinensetin, 3,5,6,7,8,3′,4′-heptamethoxyflavone, and tangeretin had no significant effect on the growth of MRC-5 cells (Figure 7b), indicating that the important PMFs had an inhibitory effect on the growth of cancer cells but a little effect on normal lung cells with no toxicity. Collectively, these results demonstrated that all the important PMFs could inhibit the proliferation of A549 cells in vitro and had no cytotoxicity to normal lung MRC-5 cells.

Further, flow cytometry assay with Annexin V-FITC/PI double staining was performed to determine whether the important PMFs could induce apoptosis in A549 cells. Compared to the control group, tangeretin and 3,5,6,7,8,3′,4′-heptamethoxyflavone significantly induced apoptosis in a dose-dependent manner at the gradient concentrations of 50 µM, 100 µM, and 200 µM, and the apoptosis induction effect of tangeretin was better than 3,5,6,7,8,3′,4′-heptamethoxyflavone. However, isosinensetin did not significantly induce cell apoptosis (Figure 7c–e). In order to explore the contribution of isosinensetin to the anticancer effect of PCR-CF during aging, an apoptosis assay was performed using the equivalent amount of flavonoid mixture (FM1, 16.5 µM tangeretin, 2.2 µM isosinensetin, and 1.7 µM 3,5,6,7,8,3′,4′-heptamethoxyflavone; FM2, 16.5 µM tangeretin and 1.7 µM 3,5,6,7,8,3′,4′-heptamethoxyflavone) corresponding to the important PMFs in 200 µg/mL PCR-C10F. As shown in Figure 7f, the apoptosis effect of FM2 showed no difference from the FM1 and PCR-C10F, implying that tangeretin and 3,5,6,7,8,3′,4′-heptamethoxyflavone were the crucial active components to predict the anticancer effect of PCR-C during storage.

Taken together, our results demonstrated that tangeretin and 3,5,6,7,8,3′,4′-heptamethoxyflavone could inhibit the cell growth and induce apoptosis in a dose-dependent manner in A549 cells. In spite of the low contents of these two PMFs in PCR-C, they showed strong anticancer effect against NSCLC. Our results were consistent with the previous report that tangeretin was the most crucial active ingredient in long-term stored citrus peel, and it could inhibit cell proliferation, induce the cell cycle arrest and cell apoptosis so as to fight against oral squamous cell carcinoma [45]. Based on these findings, we concluded that the increasing content of the PMFs during storage might improve the quality of PCR-C, and that the tangeretin and 3,5,6,7,8,3′,4′-heptamethoxyflavone might be the contributors to the improvement of the efficacy and quality of long-term stored PCR-C, and thus they might be crucial active indicators for predicting the anticancer effect of PCR-C during storage against NSCLC.

### 3.8. Crucial Active PMFs Induces ROS Accumulation in A549

**Figure 8:**
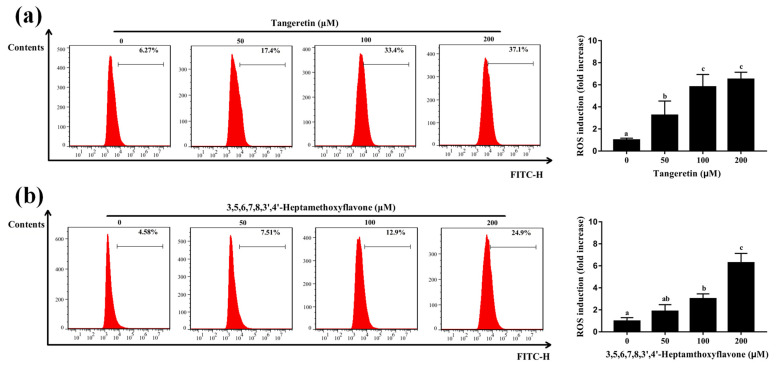
Crucial active PMFs-induced ROS accumulation of A549 cells. ROS generation was detected by flow cytometry after 24-h exposure to tangeretin (**a**) and 3,5,6,7,8,3′,4′-heptamethoxyflavone (**b**) followed by DCFH-DA staining. The fluorescence intensity ratio of the treatment group to the control group was calculated. The data were expressed as the means ± SD of the triplicates of each independent experiment. The different lowercase letters above the bars indicate a significant difference (*p* < 0.05) between the different groups through one-way ANOVA and Duncan’s multiple comparisons.

The increasing evidence indicated that ROS plays a pivotal role in mediating the viability of cancer cells [40,41]. Our data showed that ROS accumulation induced by the PCR-CF could inhibit the cell growth, induce cell cycle arrest and apoptosis in A549 cells. Therefore, ROS generation that was induced by the crucial PMFs in A549 cells was detected by flow cytometry with DCFH-DA staining. As shown in Figure 8a,b, the ROS level was dramatically increased in a dose-dependent manner when A549 cells were treated with tangeretin and 3,5,6,7,8,3′,4′-heptamethoxyflavone at the gradient concentrations (50, 100, 200 µM) for 24 h. In conclusion, our results further confirmed that the crucial PMFs induced ROS accumulation, thus possibly inhibiting the cell viability and inducing apoptosis of A549 cells.

## 4. Conclusions

In this study, cluster analysis and PCA showed that the free PMFs in PCR-C during storage fell into three clusters, namely, fresh and PCR-C01 (Cluster 1), PCR-C03, and PCR-C05 (Cluster 2), and PCR-C10 (Cluster 3). The PLS-DA results showed that three PMFs including 3,5,6,7,8,3′,4′-heptamethoxyflavone, tangeretin, and isosinensetin were identified as the important PMFs that could distinguish types of PCR-C during storage. Furthermore, our data showed that the anticancer effect against NSCLC of PCR-CF was enhanced with the extension of storage periods, and that tangeretin and 3,5,6,7,8,3′,4′-heptamethoxyflavone exhibited an anticancer effect on A549 cells, but isosinensetin failed to induce the apoptosis of A549 cells. The apoptosis induction effect of FM2 showed no difference from that of FM1 and PCR-C10F, indicating that tangeretin and 3,5,6,7,8,3′,4′-heptamethoxyflavone collectively contributed to the anticancer effect of PCR-C. Our results confirmed that tangeretin and 3,5,6,7,8,3′,4′-heptamethoxyflavone contributed to the high quality of the long-term stored PCR-C, which provides a theoretical basis for scientific utilization, rational storage, and quality control of PCR-C. However, the anticancer effect of PCR-C needs further research on other NSCLC cell lines such as H1299 and H1975 and in vivo study in the future.

## Figures and Tables

**Table 1:** The IC_50_ values of A549 cells that were treated with PCR-CF during storage.

IC_50_ Values (µg/mL)					
Samples	Fresh-F	PCR-C01F	PCR-C03F	PCR-C05F	PCR-C10F
A549	379.4	213.7	253.6	208.2	190.2

**Table 2:** The IC_50_ values of A549 cells that were treated with the important PMFs.

IC_50_ Values (µM)			
Samples	Tangeretin	Isosinensetin	3,5,6,7,8,3′,4′-Heptamethoxyflavone
A549	118.5	197.6	208.6

## Data Availability

Data is within the article.

## References

[1] Liu H., Qiu N., Ding H., Yao R. (2008). Polyphenols contents and antioxidant capacity of 68 Chinese herbals suitable for medical or food uses. Food Res. Int..

[2] Wei D., Ci X., Chu X., Wei M., Hua S., Deng X. (2012). Hesperidin Suppresses Ovalbumin-Induced Airway Inflammation in a Mouse Allergic Asthma Model. Inflammation.

[3] Tang X., Zhao H., Jiang W., Zhang S., Guo S., Gao X., Yang P., Shi L., Liu L. (2018). Pharmacokinetics and pharmacodynamics of citrus peel extract in lipopolysaccharide-induced acute lung injury combined with Pinelliae Rhizoma Praeparatum. Food Funct..

[4] Wang J., Huang Q., Zhang Y., Su J., Liu J. (2012). Current Situation of the Long-stored TCM Research and Exploration of the Ideas. China Pharm..

[5] Fu M., Xu Y., Chen Y., Wu J., Yu Y., Zou B., An K., Xiao G. (2017). Evaluation of bioactive flavonoids and antioxidant activity in Pericarpium Citri Reticulatae (Citrus reticulata ‘Chachi’) during storage. Food Chem..

[6] Luo M., Luo H., Hu P., Yang Y., Wu B., Zheng G. (2018). Evaluation of chemical components in Citri Reticulatae Pericarpium of different cultivars collected from different regions by GC-MS and HPLC. Food Sci. Nutr..

[7] Youn K., Lee S., Jun M. (2019). Discovery of Nobiletin from Citrus Peel as a Potent Inhibitor of beta-Amyloid Peptide Toxicity. Nutrients.

[8] Yao X., Zhu X., Pan S., Fang Y., Jiang F., Phillips G.O., Xu X. (2012). Antimicrobial activity of nobiletin and tangeretin against Pseudomonas. Food Chem..

[9] Bian X., Xie X., Cai J., Zhao Y., Miao W., Chen X., Xiao Y., Li N., Wu J.-L. (2022). Dynamic changes of phenolic acids and antioxidant activity of Citri Reticulatae Pericarpium during aging processes. Food Chem..

[10] Luo Y., Zeng W., Huang K.-E., Li D.-X., Chen W., Yu X.-Q., Ke X.-H. (2019). Discrimination of Citrus reticulata Blanco and Citrus reticulata ‘Chachi’ as well as the Citrus reticulata ‘Chachi’ within different storage years using ultra high performance liquid chromatography quadrupole/time-of-flight mass spectrometry based metabolomics approach. J. Pharm. Biomed. Anal..

[11] Duma N., Santana-Davila R., Molina J.R. (2019). Non-Small Cell Lung Cancer: Epidemiology, Screening, Diagnosis, and Treatment. Mayo Clin. Proc..

[12] Zarogoulidis K., Zarogoulidis P., Darwiche K., Boutsikou E., Machairiotis N., Tsakiridis K., Katsikogiannis N., Kougioumtzi I., Karapantzos I., Huang H. (2013). Treatment of non-small cell lung cancer (NSCLC). J. Thorac. Dis..

[13] Lemjabbar-Alaoui H., Hassan O.U., Yang Y.-W., Buchanan P. (2015). Lung cancer: Biology and treatment options. Biochim. Et Biophys. Acta-Rev. Cancer.

[14] Duan L., Dou L.-L., Yu K.-Y., Guo L., Chen B.-Z., Li P., Liu E.H. (2017). Polymethoxyflavones in peel of Citrus reticulata ‘Chachi’ and their biological activities. Food Chem..

[15] Fu M., Zou B., An K., Yu Y., Tang D., Wu J., Xu Y., Ti H. (2019). Anti- asthmatic activity of alkaloid compounds from Pericarpium Citri Reticulatae (Citrus reticulata ‘Chachi’). Food Funct..

[16] Chen X.-M., Tait A.R., Kitts D.D. (2017). Flavonoid composition of orange peel and its association with antioxidant and anti-inflammatory activities. Food Chem..

[17] Yu Q., Tao Y., Huang Y., Zogona D., Wu T., Liu R., Pan S., Xu X. (2022). Aged Pericarpium Citri Reticulatae ‘Chachi’ Attenuates Oxidative Damage Induced by tert-Butyl Hydroperoxide (t-BHP) in HepG2 Cells. Foods.

[18] Liang S., Wen Z., Tang T., Liu Y., Dang F., Xie T., Wu H. (2022). Study on flavonoid and bioactivity features of the pericarp of Citri Reticulatae ‘chachi’ during storage. Arab. J. Chem..

[19] Choi M.-Y., Chai C., Park J.H., Lim J., Lee J., Kwon S.W. (2011). Effects of storage period and heat treatment on phenolic compound composition in dried Citrus peels (Chenpi) and discrimination of Chenpi with different storage periods through targeted metabolomic study using HPLC-DAD analysis. J. Pharm. Biomed. Anal..

[20] Wang H., Chen G., Fu X., Liu R.-H. (2016). Effects of aging on the phytochemical profile and antioxidative activity of Pericarpium Citri Reticulatae ‘Chachiensis’. Rsc Adv..

[21] Barker M., Rayens W. (2003). Partial least squares for discrimination. J. Chemom..

[22] Zheng Y., Zeng X., Peng W., Wu Z., Su W. (2018). Study on the Discrimination between Citri Reticulatae Pericarpium Varieties Based on HS-SPME-GC-MS Combined with Multivariate Statistical Analyses. Molecules.

[23] Lloyd G.R., Stone N. (2015). Method for Identification of Spectral Targets in Discrete Frequency Infrared Spectroscopy for Clinical Diagnostics. Appl. Spectrosc..

[24] Yang M., Jiang Z., Wen M., Wu Z., Zha M., Xu W., Zhang L. (2022). Chemical Variation of Chenpi (Citrus Peels) and Corresponding Correlated Bioactive Compounds by LC-MS Metabolomics and Multibioassay Analysis. Front. Nutr..

[25] Liu X., Jiang Q., Liu H., Luo S. (2019). Vitexin induces apoptosis through mitochondrial pathway and PI3K/Akt/mTOR signaling in human non-small cell lung cancer A549 cells. Biol. Res..

[26] Ren H., Zhang Y.-Y., Li Y.-L., Bai M., Yan Q.-L., Huang X.-X., Cui W., Zhao H., Gu L., Liu Q. (2022). Semisynthesis and Non-Small-Cell Lung Cancer Cytotoxicity Evaluation of Germacrane-Type Sesquiterpene Lactones from Elephantopus scaber. J. Nat. Prod..

[27] Wang J., Li X. (2021). Chamaejasmine induces apoptosis in human non-small-cell lung cancer A549 cells through increasing the Bax/Bcl-2 ratio, caspase-3 and activating the Fas/FasL. Minerva Med..

[28] Song M., Charoensinphon N., Wu X., Zheng J., Gao Z., Xu F., Wang M., Xiao H. (2016). Inhibitory Effects of Metabolites of 5-Demethylnobiletin on Human Nonsmall Cell Lung Cancer Cells. J. Agric. Food Chem..

[29] Park K.I., Park H.S., Nagappan A., Hong G.E., Lee D.H., Kang S.R., Kim J.A., Zhang J., Kim E.H., Lee W.S. (2012). Induction of the cell cycle arrest and apoptosis by flavonoids isolated from Korean Citrus aurantium L. in non-small-cell lung cancer cells. Food Chem..

[30] Park K.-I., Park H.-S., Kim M.-K., Hong G.-E., Nagappan A., Lee H.-J., Yumnam S., Lee W.-S., Won C.-K., Shin S.-C. (2014). Flavonoids identified from Korean Citrus aurantium L. inhibit Non-Small Cell Lung Cancer growth in vivo and in vitro. J. Funct. Foods.

[31] Ma L.-L., Wang D.-w., Yu X.-D., Zhou Y.-L. (2016). Tangeretin induces cell cycle arrest and apoptosis through upregulation of PTEN expression in glioma cells. Biomed. Pharmacother..

[32] Chin-Chen C., Shih-Ying C., Charng-Cherng C., Pin-Der D. (2017). Antiproliferative effect of sweet orange peel and its bioactive compounds against human hepatoma cells, in vitro and in vivo. J. Funct. Foods.

[33] Woelfle U., Seelinger G., Bauer G., Meinke M.C., Lademann J., Schempp C.M. (2014). Reactive Molecule Species and Antioxidative Mechanisms in Normal Skin and Skin Aging. Ski. Pharmacol. Physiol..

[34] Menon S., Devi S.K.S., Santhiya R., Rajeshkumar S., Kumar V.S. (2018). Selenium nanoparticles: A potent chemotherapeutic agent and an elucidation of its mechanism. Colloids Surf. B-Biointerfaces.

[35] Bossis G., Sarry J.-E., Kifagi C., Ristic M., Saland E., Vergez F., Salem T., Boutzen H., Baik H., Brockly F. (2014). The ROS/SUMO Axis Contributes to the Response of Acute Myeloid Leukemia Cells to Chemotherapeutic Drugs. Cell Rep..

[36] Fouzder C., Mukhuty A., Kundu R. (2021). Kaempferol inhibits Nrf2 signalling pathway via downregulation of Nrf2 mRNA and induces apoptosis in NSCLC cells. Arch. Biochem. Biophys..

[37] Liang F., Fang Y., Cao W., Zhang Z., Pan S., Xu X. (2018). Attenuation of tert-Butyl Hydroperoxide (t-BHP)-Induced Oxidative Damage in HepG2 Cells by Tangeretin: Relevance of the Nrf2-ARE and MAPK Signaling Pathways. J. Agric. Food Chem..

[38] Kim H.-I., Jeong Y.-U., Kim J.-H., Park Y.-J. (2018). 3,5,6,7,8,3,4-Heptamethoxyflavone, a Citrus Flavonoid, Inhibits Collagenase Activity and Induces Type I Procollagen Synthesis in HDFn Cells. Int. J. Mol. Sci..

[39] Lee Y.Y., Lee E.-J., Park J.-S., Jang S.-E., Kim D.-H., Kim H.-S. (2016). Anti-Inflammatory and Antioxidant Mechanism of Tangeretin in Activated Microglia. J. Neuroimmune Pharmacol..

[40] Guo X.-Q., Cao Y.-L., Hao F., Yan Z.-R., Wang M.-L., Liu X.-W. (2017). Tangeretin alters neuronal apoptosis and ameliorates the severity of seizures in experimental epilepsy-induced rats by modulating apoptotic protein expressions, regulating matrix metalloproteinases, and activating the PI3K/Akt cell survival pathway. Adv. Med. Sci..

[41] Sawamoto A., Okuyama S., Amakura Y., Yoshimura M., Yamada T., Yokogoshi H., Nakajima M., Furukawa Y. (2017). 3,5,6,7,8,3′,4′-Heptamethoxyflavone Ameliorates Depressive-Like Behavior and Hippocampal Neurochemical Changes in Chronic Unpredictable Mild Stressed Mice by Regulating the Brain-Derived Neurotrophic Factor: Requirement for ERK Activation. Int. J. Mol. Sci..

[42] Lin J.-J., Huang C.-C., Su Y.-L., Luo H.-L., Lee N.-L., Sung M.-T., Wu Y.-J. (2019). Proteomics Analysis of Tangeretin-Induced Apoptosis through Mitochondrial Dysfunction in Bladder Cancer Cells. Int. J. Mol. Sci..

[43] Iwase Y., Takemura Y., Ju-ichi M., Yano M., Ito C., Furukawa H., Mukainaka T., Kuchide M., Tokuda H., Nishino H. (2001). Cancer chemopreventive activity of 3,5,6,7,8,3′,4′-heptamethoxyflavone from the peel of citrus plants. Cancer Lett..

[44] Chen Y.-K., Wang H.-C., Ho C.-T., Chen H.-Y., Li S., Chan H.-L., Chung T.-W., Tan K.-T., Li Y.-R., Lin C.-C. (2015). 5-Demethylnobiletin promotes the formation of polymerized tubulin, leads to G2/M phase arrest and induces autophagy via JNK activation in human lung cancer cells. J. Nutr. Biochem..

[45] Yu Z., Wu Y., Ma Y., Cheng Y., Song G., Zhang F. (2022). Systematic analysis of the mechanism of aged citrus peel (Chenpi) in oral squamous cell carcinoma treatment via network pharmacology, molecular docking and experimental validation. J. Funct. Foods.

